# Ensuring generalized fairness in batch classification

**DOI:** 10.1038/s41598-023-45943-1

**Published:** 2023-11-02

**Authors:** Manjish Pal, Subham Pokhriyal, Sandipan Sikdar, Niloy Ganguly

**Affiliations:** 1 Department of Computer Science and Engineering, IIT-Kharagpur, Kharagpur, 721302 India; 2 Department of Computer Science and Engineering, IIT-Ropar, Ropar, 140001 India; 3 L3S Research Center, Leibniz University of Hannover, 30167 Hannover, Germany

**Keywords:** Computational science, Computer science

## Abstract

In this paper, we consider the problem of batch classification and propose a novel framework for achieving fairness in such settings. The problem of batch classification involves selection of a set of individuals, often encountered in real-world scenarios such as job recruitment, college admissions etc. This is in contrast to a typical classification problem, where each candidate in the test set is considered separately and independently. In such scenarios, achieving the same acceptance rate (i.e., probability of the classifier assigning positive class) for each group (membership determined by the value of sensitive attributes such as gender, race etc.) is often not desirable, and the regulatory body specifies a different acceptance rate for each group. The existing fairness enhancing methods do not allow for such specifications and hence are unsuited for such scenarios. In this paper, we define a configuration model whereby the acceptance rate of each group can be regulated and further introduce a novel batch-wise fairness post-processing framework using the classifier *confidence-scores*. We deploy our framework across four real-world datasets and two popular notions of fairness, namely *demographic parity* and *equalized odds*. In addition to consistent performance improvements over the competing baselines, the proposed framework allows flexibility and significant speed-up. It can also seamlessly incorporate multiple overlapping sensitive attributes. To further demonstrate the generalizability of our framework, we deploy it to the problem of *fair gerrymandering* where it achieves a better fairness-accuracy trade-off than the existing baseline method.

## Introduction

Machine learning algorithms are being increasingly deployed for decision-making in critical situations which can have a profound impact on society and human lives. It is hence important to ensure that the decisions are not biased toward particular groups characterized by sensitive attributes such as gender, ethnicity, disability etc. Recent years have witnessed significant progress in terms of designing both methods for enhancing fairness as well as metrics for evaluating fairness, with fair classification^1–15^, fair ranking^16–20^ and fair subset selections^21–23^ as important subtasks. For fair classification methods, the goal is to not only achieve high accuracy on the test set, but also fairness measured in terms of metrics such as demographic parity and equalized odds^24^. While demographic parity ensures that the acceptance rates (i.e., probability of the classifier assigning positive class) are the same for all subgroups, equalized odds requires the same in terms of true positive rates (TPR) and false positive rates (FPR). Both fair ranking and fair subset selection problem involve selecting a set of candidates from a large pool such that the overall utility is maximized while ensuring fairness. Fair ranking additionally requires the obtained set to be ordered by decreasing utility.

### Batch classification

For a large class of problem settings like recruitment, college admission etc., a set of agents (candidates) are selected simultaneously; we refer to this problem as *batch-classification*. This paradigm is different from the term *batch-wise classification* has been discussed in papers like^25^ in which it is defined to mean samples in the same batch are learned and classified collectively. We perform the task of classifying and ensuring the fairness of an entire batch together during *test* time. More specifically, we consider batch classification as a post-processing step where the entire test set is provided and the classification needs to be performed collectively over the entire test set. This is contrary to traditional classification set-up which typically is a point-wise task where each element in the test set is labelled independently of other elements in the test set. There are DNN based algorithms like^7,10^ that rely on batch-wise training, however, here we are talking about batchwise inference during *test* time. Despite the apparent similarities to classification, ranking and subset selection, batch classification does not require the relevance/utility score required by ranking and subset selection algorithms.

**Figure 1:**
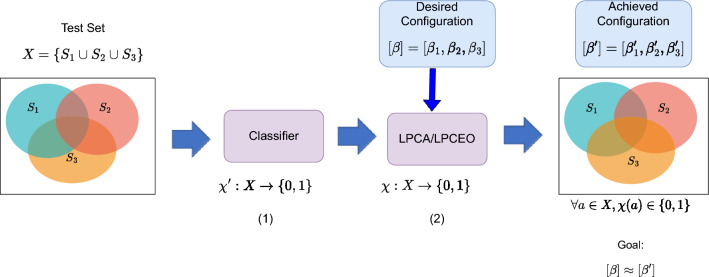
The proposed framework for fair batch classification. We are given a text set *X*. *X* consists of subgroups - $S_1$, $S_2$ and $S_3$ representing a particular value of a certain sensitive attribute. For example, $S_1$ can represent all individuals with gender male, $S_2$ could represent all black people etc.. Any item *a* in *X* can belong to one or more subgroups. We are also provided with a configuration $[\beta ] = [\beta _1, \beta _2, \beta _3]$ representing the acceptance rate corresponding to the subgroups $S_1$, $S_2$ and $S_3$. In the first step (1), we deploy a classifier model to obtain a labeling ($\chi '$) for each item in the test set. (2) We then deploy a linear programming based framework (LPCA/LPCEO) which takes as input $\chi '$ and $[\beta ]$ to obtain another labeling $\chi$ which achieves a configuration $[\beta ^{'}_1, \beta ^{'}_2, \beta ^{'}_3]$ such that $\forall i, \beta ^{'}_{i} \approx \beta _i$. To ensure low demographic disparity we would like all $\beta _i$’s to be same.

### Limitations of existing methods

 The general notion of fairness inherently assumes similar acceptance rates across groups. We posit that fairness is a concept bounded by social conditions, e.g. in societies where female representation is minimal, a law ensuring (say) 30% participation of women in public positions can be considered fair and progressive. Hence, any fairness-enhancing framework needs to be independent of any underlying ‘social’ assumption and should be able to take the social constraints as input. To the best of our knowledge, most of the existing frameworks are unsuited for such a scenario.

### Goals and approach

 Consequently, in this paper, we consider the problem of fair batch classification, where the acceptance rate of each group is taken as input from the user. The goal is to come up with a classification that achieves the desired acceptance rate for each group. To this aim, we first define the configuration model whereby the desirable acceptance rate for each group can be provided as input to the algorithm. We further demonstrate that the two popular metrics for evaluating fairness – demographic parity and equalized odds – can be reformulated in terms of acceptance rates. In order to deal with the configuration model, we consider each group as a set and develop a linear programming based solution which minimizes demographic parity and equalized odds while maintaining accuracy. The proposed framework can also seamlessly incorporate multiple overlapping sensitive subgroups. We illustrate our framework in Fig. 1.

### Results and contributions

 We perform experiments on several real-world datasets with both demographic parity and equalized odds as fairness criteria. Our proposed framework consistently outperforms the state-of-the-art fair classification, ranking and subset selection methods across several real-world and synthetic datasets. Beyond decent performance gains, our framework can also adapt to any given configuration and is capable of dealing with multiple overlapping subgroups. To further demonstrate its wider applicability, we deploy our framework to a related problem of fair gerrymandering. where it outperforms the existing baseline.

## Prior and related works

The fair classification algorithms in the existing literature can be broadly classified into three groups: pre-processing, in-processing and post-processing based algorithms.

### Pre-processing

The goal is to pre-process the training data such that any classification algorithm trained on this data would generate unfairness-free outcomes. This is usually done by generating fair representations as is done by Feldman et al.^2^, Dwork et al.^26^, Kamiran and Calders^1^, Edwards and Storkey^27^, Madras et al.^28^, Beutel et al.^29^, Ruoss et al.^8^ Rodriguez et al.^30^ and Zhao et al.^31^.

### In-processing

Here the idea is to add constraints for fairness as regularizer to the training objective function for optimization; examples—Calders and Verwer^32^, Kamishima et al.^33^, Bechavod and Ligett^34^ Bilal Zafar et al.^6^, Agarwal et al.^5^, Wu et al.^35^, Padala and Gujar^36^, Zhang et al.^37^, Yurochkin et al.^38^, Celis et al.^15^, Roh et al.^39^, Yang et al.^14^, Cho et al.^10^, Romano et al.^13^, Mary et al.^11^.

### Post-processing

The third and final strategy consists of first running a standard classifier like SVM or Logistic-regression on the training data and then using the model to mitigate unfairness in the test data. This approach has been used by Hardt et al.^12^; Corbett-Davis et al.^40^; Agarwal et al.^5^ Narasimhan^41^ and Wei et al.^42^.

From the perspective of fair ranking algorithms, relevant algorithms include Celis et al.^20^, Singh et al.^18^, Zehlike et al.^17^ and Zehlike et al.^16^. While the last one is an in-processing learning to rank algorithm, others are re-ranking algorithms without a train-test data. Fair subset selection, although has been predominantly studied in the streaming setting, there are some algorithms which have focussed on the static setting like Mehrotra et al.^23^. The Greedy-fair algorithm mentioned in the paper is based on an old paper by Nemhauser et al.^43^, that has been revisited, in the context of demographic parity, in Halabi et al.^44^. Since fair batch classification has not been studied per se in the literature, we compare our algorithms with best performing fair classification algorithms, fair ranking and subset selection algorithms. From the very definition of fair batch classification, it is clear that any fair classification algorithm is also a fair batch classification algorithm. Hence, we can consider any fair classification algorithm as a baseline for comparison. In contrast, fair ranking and subset selection algorithms need to be adapted to make meaningful comparison with our batch classification algorithms.

#### Distinction from other post-processing algorithms

 We would like to emphasize that our algorithms (**LPCA** and **LPCEO**) despite being linear program (LP) based post-processing, are quite different from other randomized post-processing algorithms like Agarwal et al.^5^ and Hardt et al.^12^ which apply linear constraints for ensuring DP and EO. In Agarwal et al., the authors use a constant (independent of the size of training and test data) dimensional linear constraint space to find a *threshold* $\tau$ that can be used on the class probabilities (referred to as confidence-scores) of the base classifier to predict classes. The overall optimization problem is not linear, and it relies on *cost sensitive classification algorithm* paradigm. In a similar vein, the LP used by Hardt et al.^12^ is also low dimensional (4 variables and 3 constraints for binary sensitive attribute) and finds a threshold to decide the classes. In contrast, our batch classification algorithms are deterministic (not considering the base classifier) and LPs are of size $n \times m$ where *n* is the number of examples in the test set and *m* is the total number of subpopulations in the dataset (e.g. $m=2$ for single binary sensitive attribute). We use the class probabilities of the base classifier only for the test set. There are also post-processing algorithms which transform the confidence score of a base classifier^42^, but their optimization problem is not linear and makes several theoretical assumptions on the datasets. Finally, unlike the algorithms discussed above, the results generated by our algorithm are not sensitive to changes in the distribution of sensitive attributes in the training data.

## Method

### Fairness evaluation metrics

We first provide a brief overview of two fairness metrics - demographic parity (DP) and equalized odds (EO) which we use for our study. These are the two most widely used metrics in the literature and represent two different categories of fairness metrics, namely independence and separation^24^. We also formally introduce the configuration model. We further reformulate DP and EO in terms of the configuration model.

#### Demographic parity

Demographic parity is originally defined considering that the positive group selection rate (which we will refer to as *acceptance rate*) needs to be the same across the two groups of a binary sensitive attribute. This measure is also referred to as statistical parity in the literature.

Let the population size be *n*, then in case of single binary sensitive attribute, the set of subpopulations corresponding to each sensitive value can be represented as S = $\{S_1,S_2\}$ where $S_1 \cap S_2 = \phi$ and $S_1 \cup S_2 = n$. $\hat{Y}={0, 1}$ represents the inferred labels. Demographic Disparity (DDP) [Single] can be accordingly defined as

$$\begin{aligned} DDP_S= & {} {\bigl |\mathbb {P}[\hat{Y} = 1|S=S_2] - \mathbb {P}[\hat{Y} = 1| S=S_1]\bigr |}, \end{aligned}$$

and the system is deemed to have demographic parity when the value is close to zero. The $\mathbb {P}[\hat{Y} = 1]$ refers to the probability of being selected in the positive group.

The definition can further be extended to multi-attribute and multivalued case. Let there be *k* attributes, with the $i^{th}$ attribute assuming $m_i$ values, then *m* (= $\sum _{i=1}^k m_i$) is the total number of sensitive values the population can assume. Correspondingly, $S = \{S_1,S_2 \dots S_m\}$ is the set of subpopulations each representing a sensitive value. Unlike in the previous case, here the subpopulations are not mutually exclusive. Here Demographic Disparity (DDP) [Multiple] can be defined as^6,7^.

$$\begin{aligned} DDP_M= & {} \bigl |\max \limits _{j} \mathbb {P}[\hat{Y} = 1|S=S_j] - \min \limits _{j}\mathbb {P}[\hat{Y} = 1|S=S_j] \bigl |.\end{aligned}$$

#### Equalized odds

Hardt et al.^12^ propose the notion of equalized odds for single binary sensitive attribute $S=\{S_1, S_2\}$ and ground-truth labeling $Y=\{0, 1\}$ based on which we define Difference of Equalized Odds (DEO) [Single] as follows:

$$\begin{aligned} DEO_S&=  (\mathbb {P}[\hat{Y} = 1| Y=1, S=S_1] - \mathbb {P}[\hat{Y} = 1| Y=1, S=S_2]) \\&\quad +   (\mathbb {P}[\hat{Y} = 1| Y=0, S=S_1] - \mathbb {P}[\hat{Y} = 1| Y=0, S=S_2]) \end{aligned}$$

This definition essentially stipulates that the True Positive Rate ($TPR_i$ = $\mathbb {P}[\hat{Y} = 1| Y=1, S=S_i]$) and the False Positive Rate ($FPR_i$ = $\mathbb {P}[\hat{Y} = 1| Y=0, S=S_i]$) be the same across all the subpopulations ($\{S_1, S_2\}$) of a given sensitive attribute *S*. To generalize the definition of difference of equalized odds to multiple sensitive attributes we define Difference of Equalized Odds (DEO) [Multiple] for multiple sensitive attributes as follows

$$\begin{aligned} DEO_M= & {} \left(\max \limits _j TPR_j - \min \limits _j TPR_j\right) + \left(\max \limits _j FPR_j - \min \limits _j FPR_j\right). \end{aligned}$$

Let $DTPR_M = (\max \limits _j TPR_j - \min \limits _j TPR_j)$ be the Difference of *TPR* and $DFPR_M = (\max \limits _j FPR_j - \min \limits _j FPR_j)$ be the Difference of *FPR*, then $DEO_M = DTPR_M + DFPR_M$. We can easily observe that there is a trivial solution to achieve zero $DEO_M$. Simply assign label 1 to all the records. But this trivial solution may lead to very low accuracy. Similarly a uniformly random 0/1 labelling also leads to zero $DEO_M$ on expectation. Instead, we are looking for labellings that lead to low $DEO_M$ and high accuracy.

#### Configuration model

A configuration (‘config’ in short) refers to the acceptance rates [${\beta }$] = $\{ \beta _1, \beta _2 \cdots \beta _{m}\}$ corresponding to each subpopulation. Normally, it is assumed the acceptance rate is same for all sub-population. However, in real life this may not be the case; in many cases policymakers may decide to have different acceptance rates for different subpopulation.

##### Connection to demographic parity

 We can assume that there is a desirable [${\beta }$] which is taken as input by an algorithm and the algorithm outputs a [${\beta '}$].

We can assume that there is a desirable [${\beta }$] which is taken as input by an algorithm and the algorithm outputs a [${\beta '}$] which is achieved. The Demographic DisParity [Configuration] in such a setting can be defined as

1 $$\begin{aligned} DDP_C ([\varvec{\beta }], [\varvec{\beta '}])&= \Vert [\varvec{\beta }] - [\varvec{\beta '}]\Vert _{\infty } = \max _{j} |\beta _{j} - \beta _{j}'| .\end{aligned}$$

The equation measures how much deviation a particular configuration has gone through w.r.t. the desirable condition. Under certain constraints, one can establish a relation between $DDP_M$ and $DDP_C$

###### Lemma 1

Given a configuration [$\beta$] in which the acceptance rates across all the subpopulation is same and equal to $\beta$, one can show $DDP_M$([$\beta '$])/2 $\le$ $DDP_C$([$\beta$], [$\beta '$]) where [$\beta '$] = $\{\beta '_1, \beta '_2, \dots , \beta '_m\}$ is an arbitrary configuration and $\beta \in [\min \limits _j \beta '_j, \max \limits _j \beta '_j]$.

###### Proof

Under the condition that $\beta \in [\min \limits _{j} \beta '_{j}, \max \limits _{j} \beta '_{j}]$, we can write $DDP_M(\varvec{\beta '}) = \max \limits _{j} \beta '_{j} - \min \limits _{j} \beta '_{j} = (\max \limits _{j} \beta '_{j} - \beta ) + (\beta - \min \limits _{j} \beta '_{j}) \le 2 \cdot \max \limits _{j} |\beta '_{j} - \beta | = 2 DDP_C([\varvec{\beta }], [\varvec{\beta '}])$.

##### Connection to equalized odds

 If one dissect the definition of Equalized Odds, it has the notion of configuration inbuilt in the definition. According to configuration model, we had defined $\beta _j = \mathbb {P}[\hat{Y} = 1| S=S_j]$. Using Bayes rule of conditional probability we can show the dependence of $TPR_j$ and $FPR_j$ on $\beta _j$ as

$$\begin{aligned} \mathbb {P}[\hat{Y} = 1| Y=1, S=S_j]= & {} \mathbb {P}[\hat{Y} = 1| S=S_j] \cdot \Delta _j \\ \mathbb {P}[\hat{Y} = 1| Y=0, S=S_j]= & {} \mathbb {P}[\hat{Y} = 1| S=S_j] \cdot \Gamma _j \end{aligned}$$

where $\Delta _j = \frac{\mathbb {P}[Y = 1| \hat{Y}=1, S=S_j]}{\mathbb {P}[Y=1 |S=S_j]}$. and $\Gamma _j = \frac{\mathbb {P}[Y = 0| \hat{Y}=1, S=S_j]}{\mathbb {P}[Y=0 |S=S_j]}$ Similarly we can write the $FPR_j$ in terms of $\beta _j$ as well and hence can write $DEO_M$ as

$$\begin{aligned} DEO_M= & {} (\max \limits _j \beta _j \cdot \Delta _j - \min \limits _j \beta _j \cdot \Delta _j) + (\max \limits _j \beta _j \cdot \Gamma _j - \min \limits _j \beta _j \cdot \Gamma _j). \end{aligned}$$

The linking of a configuration with $DEO_M$ allows us to compare two competing algorithms whereby we measure the $DEO_M$ and accuracy achieved by two algorithms at the same configuration [$\beta$]. In the above formulation, unlike *DDP*, we use $\beta _j$’s only as a tool to compare baselines wherein we use a particular configuration and measure the corresponding *DEO* and accuracy. and hence we do not address the issue of input and output (from an algorithm) configurations and assume them to be the same. Note that fixing a configuration [$\beta$] and minimizing $DEO_M$ doesn’t necessarily mean that we are trying to satisfy both DP and EO criteria together (which are otherwise known to be incompatible^45^).

### Batch classification

#### Objective

 Given a test set *X* with $|X| = n$, and a collection $S=\{S_1,S_2,\dots ,S_m\}$ of subsets of *X*, representing subpopulations across the sensitive attributes and a configuration $[\beta ]= (\beta _1, \beta _2, \dots , \beta _m)$, obtain a labeling $\chi = \{0, 1\}$ of the items in *X* such the configuration $[\beta ]$ could be realized for all the *m* (possibly overlapping) subsets. While acceptance rates directly maps to demographic parity (lemma 1), for DEO additional constraints on *TPR* and *FPR* for each subset is provided as input that needs to be satisfied along with the configuration.

#### Decidability problem

As a first step, we investigate the simpler decidability version of the above objective. Formally, decision problem corresponding to demographic disparity and equalized odds can be defined as:

##### Problem 1

(Demographic Disparity) Let *X* be a universal set with $|X| = n$. Given a collection $S = \{S_1,S_2, \cdots S_m\}$ of subsets of *X* as the set of all subpopulations across the sensitive attributes and a configuration [${\beta }$] $= (\beta _1,\beta _2, \dots \beta _m)$, decide whether there exists a 0/1 labelling of elements of the universal set *X* such that the configuration [${\beta }$] can be realized for all the *m* (possibly overlapping) sets.

##### Problem 2

(Equalized Odds) Let *X* be a universal set with $|X| = n$, such that each element $x \in X$ has a tag $r(x) \in \{0,1\}$. Given a collection $S = \{S_1,S_2, \cdots S_m\}$ of subsets of *X* as the set of all subpopulations across the sensitive attributes, a configuration [${\beta }$] $= (\beta _1,\beta _2, \dots \beta _m)$ and (*tpr*, *fpr*), decide whether there exists a 0/1 labelling of the elements of *X* such that the configuration can be realized with the given *tpr* and *fpr* for all the *m* sets w.r.t the given tags *r*(.) .

In certain formulations of the problem of ensuring fairness when posed as a ranking and set-selection problem^20,22,23^, the problem is known to be **NP**-hard. Due to the possibly non-polynomial time solutions to these problems, we now proceed to devise a more practical LP solution of the original objective of obtaining a labeling of the elements in a test set that achieves the provided configuration as well as satisfying any additional fairness constraints.

#### Linear programming based solution

In this section, we step-by-step propose the LP solution. At first we assume that the data is untagged (i.e. without ground truth label) and propose an LP to reduce *DDP*. This LP can’t be used to reduce *DEO* as the *EO* criterion inherently needs data to be tagged. Next, we consider the data to be tagged (with ground truth label) and write an LP that can be used to reduce the *DDP* and *DEO* while ensuring high accuracy.

##### Batch classification of items without tag

 In this setting, we consider a setting where the ground-truth labels of the elements to be classified are not known. Formally, given a test dataset *X* with $|X| = n$ items, the set of subpopulations belonging to all the sensitive attributes $S = \{S_1,S_2 \dots S_m\}$, find a 0/1 labelling $\chi$ of the items such that the positive (label 1) fraction for the subpopulation $S_{j}$ be $\beta _{j}$, where $\beta _{j}$ $\in$ $[\varvec{\beta }]$, is a given configuration.
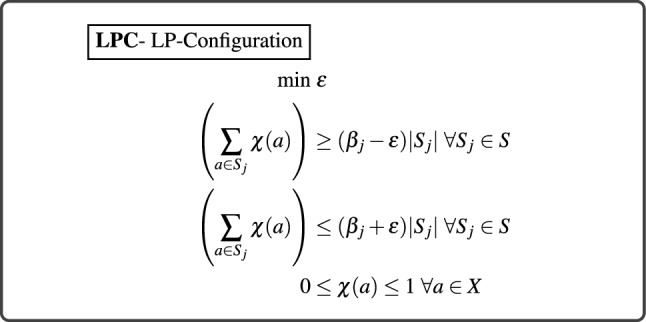


We propose an LP-solution to solve the above problem as **LPC**. In **LPC** (**L**inear **P**rogramming framework with **C**onfiguration), $\chi (a)$ represents the labelling of the item *a* and when the LP is feasible it will ensure that the acceptance rates of all values of the sensitive attributes be between $\beta _{j} - \varepsilon$ and $\beta _{j} + \varepsilon$. According to this notation, $\chi : X \rightarrow \{0,1\}$ is a function and we treat $\chi (a)$ as a variable in the linear program. As per the configuration model [$\beta$] = $\{\beta _1, \beta _2, \dots , \beta _m\}$ is input to the **LPC**, and [$\beta$’] = $\{\beta '_1 = \beta _1 + {\hat{\varepsilon }}_{1}, \beta '_2 = \beta _2 + {\hat{\varepsilon }}_{2}, \dots , \beta '_m = \beta _m + {\hat{\varepsilon }}_{m}\}$ is the output. The $\max \limits _{j} {\hat{\varepsilon }}_{j}$ = $\varepsilon$ represents $DDP_C$. With the above formulation, we deploy a standard solver (PuLP) to obtain a solution i.e., a labeling of the elements $\chi$ which satisfies the configuration constraint.

##### Batch classification of items with tag

While LPC achieves the provided configuration, for a more realistic setting the elements are tagged with a ground-truth class and a model needs to satisfy additional performance and fairness constraints based on the ground-truth. Formally, given a test dataset *X* ($|X| = n$) with each item $x_i$ associated with a ground-truth $y(x_i) \in \{0, 1\}$ and classifier prediction $\hat{y}(x_i) \in \{0, 1\}$, a set of subpopulations belonging to all the sensitive attributes $S = \{S_1,S_2 \dots S_m\}$, find a 0/1 labeling $\chi$ of the items ensuring - (1) the accuracy $\frac{1}{n}\sum _i \chi (x_i)y(x_i)$ is maximized, (2) the given configuration $[\beta ]$ is achieved, and (3) $TPR_j$ ($FPR_j$) w.r.t. *y*(.) are same for all subpopulations $S_j \in S$ (DEO constraint). In the following, we describe a LP based solution to this problem and describe in detail how the above three constraints are ensured.

**Figure 2:**
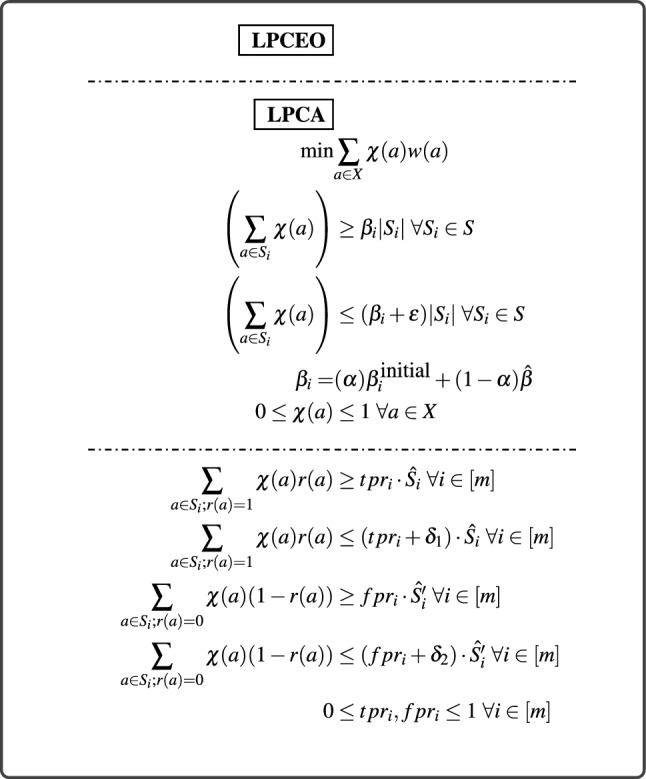
In the proposed framework, **LPCA** is the LP between dotted lines defined on the test records $a \in X$ with inputs as, target configuration (with zero disparity): $\hat{\beta }$, initial configuration: $[\beta _i^{\text{ initial }}]$, fairness tuning parameter: $\alpha$ and weights derived from confidence scores on test data: *w*(*a*), sensitive subpopulations $S_i$ in the test set and variables as $\chi (a)$. The entire linear program, **LPCEO** has additional inputs as classifier labels on test data: *r*(*a*), the number of positives (negatives) in the population $S_j$ according to the classifier: $\hat{S_i}(\hat{S_i'})$, error allowed in TPR (FPR): $\delta _1(\delta _2)$ and variables as $tpr_i,fpr_i$ which can also be user defined.

We propose **LPCA** (**L**inear **P**rogramming framework with **C**onfiguration and **A**ccuracy) and **LPCEO** (**L**inear **P**rogramming framework with **C**onfiguration and **E**qualized **O**dds), presented in Fig. 2, which try to satisfy the two constraints (in case of DDP) and equalized odds constraints (in case of DEO). Note that achieving the same acceptance rate for all the subpopulations is equivalent to achieving DDP which makes DDP a special case of configuration model. The basic structure of **LPCA** and **LPCEO** is given below. **LPCA** is the linear program that doesn’t contain the constraints corresponding to *tpr*, *fpr* variables. If we add the constraints that involve the variables *tpr* and *fpr*, we obtain **LPCEO** that can be used to ensure the equalized odds fairness constraint. The proposed LP can be considered to be a generalization of the LP relaxation studied by Mehrotra et al.^23^.

1. *Maximizing Accuracy.* Each element in the set is assigned a binary $\{0, 1\}$ label. Hence to attain higher accuracy while choosing members of one group (say female), primarily those members who have acceptance tags ($y(x_i) = 1$) need to be chosen. However, note the ground truth *y* is **not** known while the selection is made. So the tags are estimated (predicted) using a classifier, better the classifier, better the estimation. The classifier besides inferring the class (tag) of each point also returns a **confidence value**, we leverage the understanding that the classification error would minimize if one chooses the items which have been classified with higher confidence values^1,46^ to maximize the accuracy. More specifically, we derive a weight $w_{j}(a)$ from the confidence value (rank) for every item *a* in the test data that depends on the subpopulation *j* (like male, black etc.) and minimize $\sum \limits _{j=1}^{m} \sum \limits _{a \in S_{j}} \chi (a) w_{j}(a)$. We put $w_{j}(a)$ = $r_{j}$ i.e. the rank (in descending order of confidence values predicted by the classifier, in the subpopulation $S_{j}$) of the element *a*. To bring in uniformity $w_{j}(a)$ is normalized with the size of the group, hence $w_{j}(a) = \alpha _{j} \cdot \frac{r_{j}}{|S_{j}|}$, where $\alpha _{j}$ is a hyperparameter and $|S_{j}|$ is the number of elements present in that group.
2. *Achieving the desired configuration.* We provide [$\beta$] as a hard constraint along with a small value $\varepsilon$ as a relaxation. (Again experimental results show that in most of the cases, the output deviation from [$\beta$] is smaller than $\varepsilon$.) Note that unlike **LPC**, in **LPCA** and **LPCEO**, we put the single sided error, it represents the two-sided phenomenon if we assume ${\hat{\beta }}_{j}$ is equal to $\beta _{j} - \varepsilon /2$.
3. *Achieving Low* $DEO_M$. In **LPCEO**  the variables $tpr_i, fpr_i$ correspond to the TPR and FPR of the subpopulation $S_i$ and $\hat{S_i} (\hat{S'_i})$ is an approximate number of 1s (0s) in the test data in the subpopulation $S_i$ (based on classifier’s prediction). Since we do not have access to *y*(.), we use *r*(.) that is the tag returned by a chosen classifier as a proxy to *y*(.). Here $\delta _1, \delta _2$ are tunable parameters which control the $DEO_M$ of the output configuration $[\varvec{\beta }_\chi ]$. More specifically, $DEO_M([\varvec{\beta }_{\chi }]) \le \delta _1 + \delta _2$.

The accuracy of **LPCA** and **LPCEO** depends on the performance of the underlying classifier. In this paper, we assume the base classifier to be a Random Forest (RF) and provide our results according to its *confidence scores*. Similar results are obtained with Logistic Regression, SVM or a DNN based classifier. All results in which comparison with certain baselines was made are obtained using a fixed 70% / 30% train / test split of all datasets. We show the robustness of our results We again deploy PuLP to obtain a solution to the LPs.

##### Time complexity

 As mentioned before our batch processing algorithms are post-processing hence they involve two steps, a classifier training step and then a post processing step. Thus the overall convergence time of the algorithm can be written as ${{\mathcal {T}}}_{classifier} + {{\mathcal {T}}}_{LP}$ where $\mathcal{T}_{classifier}$ is the running time of the chosen classifier and ${{\mathcal {T}}}_{LP}$ is the complexity of our batch processing step. If there are *m* overall sub-populations and the size of the test set is *n*, then for both **LPCA** and **LPCEO**  using the best known theoretical LP solvers will lead to a convergence time of $\mathcal{T}_{classifier} + \tilde{O}(\sqrt{m}(mn + m^2))$^47^ where $\tilde{O}$ hides polylogarithmic factors. Since for most practical scenarios, the number of subpopulations *m* is a small constant, the convergence time can be rewritten as $\mathcal{T}_{classifier} + \tilde{O}(n)$. Thus the running time of our algorithms is dominated by the classifier’s training time.

## Results

### Experimental setup

In this section, we describe the experimental setup that includes a description of various datasets, baselines, performance metrics and configuration generation to compare with baselines.

####  Datasets

In our study we have used four real datasets namely Adult^48^, Bank^49^, COMPAS (ProPublica Recidivism)^50^, German^51^ and a synthetic dataset for evaluating the performance of the algorithms. The number of instances and classes in each attribute is written within the bracket below. First we describe the real world datasets as follows:

*Adult* (48,842 examples) Here the task is to predict whether someone makes more than $50k per year, with gender(2) and race(5) as the protected attribute.

*Bank* (41,188 examples) Each example has 20 features and the target variable is whether the client has subscribed to the term deposit service or not. We have taken age group(2) and marital status(4) (MS) as the sensitive attributes.

*ProPublica recidivism * (7,918 examples) In ProPublica’s COMPAS recidivism data, the task is to predict recidivism from someone’s criminal history, jail and prison time, demographics, and COMPAS risk scores, with race(2) and gender(2) as the protected attributes.

*German* (1,000 examples) The German credit dataset contains attributes such as personal status and sex, credit score, credit amount, housing status etc. It can be used in studies about gender inequalities on credit-related issues. The sensitive attribute being gender(2) and age(2).

*Synthetic Datasets* (8,000 examples) We generate datasets using a Python function called *sklearn.datasets.make_classification*^52^ with n_samples=8000, n_features=20, n_classes=2. We use the last *k* features as sensitive attributes where *k* is varied from 2 to 10, by converting them to 0/1 based on the condition $(\le 0)/(> 0)$. The natural positive rates vary across groups and produce non-zero DDP for all the sensitive features.

#### Baselines

We compare our proposed method with existing fair classification, fair ranking and fair subset selection methods. In specific, we consider methods that aim to achieve demographic parity and equalized odds in classification, ranking and subset selection setting.

##### Demographic parity

 When selecting appropriate fair classification baselines, we particularly consider those that can tackle both multiple as well as multivalued sensitive attributes. These include - (a). Agarwal et al.^5^, (b). Zafar et al.^6^, (c). Padala et al.^36^, (d). Yang et al,^14^ and (e) Madras et al.^53^ [The algorithms of Madras et al. only work for single binary sensitive attribute]. Similarly, for ranking **DELTR**^16^ (Disparate Exposure in Learning to Rank) and for subset selection LP-relaxation studied in Mehrotra et al.^23^ and the Greedy-Fair algorithm in fair submodular maximization described in Halabi et al.^44^ are chosen as baselines [Certain adjustments had to be made in order to adopt these methods to our setting.].

##### Equalized odds

 We only include classification baselines as the existing ranking or subset selection methods are not designed for equalized odds - (a). Agarwal et al.^5^, (b). Zafar et al.^6^, (c). Padala et al.^36^, (d). Romano et al.^13^, (e) Cho et al.^10^, (f) Mary et al.^11^ and (g) Hardt et al.^12^.

#### Performance metrics

Besides measuring the fairness metrics (here DDP and DEO), one of the standard metrics used to measure the performance is accuracy. However, we can further divide the performance with respect to the groups which gain or loss due to the fair *batch classification* process. Therefore, an important metric would be **precision**, that is the fraction of elements chosen with a positive tag in the accepted set if the number of positive (tag) elements present is more than the accepted elements. Similarly, if the number of elements to be accepted is more than the total number of positive (tag) elements present, then one needs to consider **recall**, that is, the efficacy of an algorithm depends upon the fraction of the positive elements being accepted. Since, the definition of EO has the notion of TPR (recall) and FPR (that is in turn related to precision) inbuilt in it, we don’t consider precision, recall comparisons in case of EO.

Given a particular $[\varvec{\beta }] = \{\beta _1, \beta _2, \dots , \beta _m \}$, there will be a subset of subpopulation $[\varvec{S^+}]$ whereby the natural acceptance rate (say $\beta ^{natural}_j$) of a subpopulation $S_j \in [S^+]$ be more than $\beta _j$ and likewise, for the complementary subset of subpopulation $[\varvec{S^-}]$, it would be less. We need to calculate the precision (recall) of individual subpopulations in the set $[\varvec{S^+}]$ ($[\varvec{S^-}]$). However, to avoid generating too many values, we propose the notion of **weighted precision** and **weighted recall**. Let $[S^+]~=~\{S_{1}, S_{2}, \dots , S_{k}\}$ be the subpopulations whose populations are $n_{1}, n_{2}, \dots , n_{k}$ and individual precisions are $p_{1}, p_{2}, \dots , p_{k}$, we define weighted precision of [$S^+$] as $\sum _t p_{t} n_{t} / \sum _t n_{t}$. Analogously we can define the notion of weighted recall.

#### Deriving a configuration

While for **LPC**, **LPCA** and **LPCEO**, any arbitrary configuration can be specified as input, this is not the case for the baseline algorithms where the goal is to achieve a configuration where the acceptance rates of the subpopulations are equal. Additionally, the acceptance rates achieved by the baselines need not be same as the ones specified as input. To make more fair comparison with baselines and to ensure user flexibility, we propose to deploy a tunable parameter $\alpha$ to generate configurations. Let’s assume that there is an initial configuration $[\varvec{\beta }^{\text{ initial }}]$ which can be any arbitrary input provided by the user and the acceptance rates of the subpopulations need not be same. $\hat{\beta }$ be a target configuration (e.g., achieved by a baseline algorithm) where the acceptance rates are same for all the subpopulations. We deploy $\alpha$ to downward interpolate the acceptance rate of a subpopulation for which the acceptance in $[\varvec{\beta }^{\text{ initial }}]$ is higher than $\hat{\beta }$ and vice versa. In **LPCA**  the DDP at certain $\alpha$ can be written as $DDP_M([\varvec{\beta }_{\alpha }]) = \alpha DDP_M([\varvec{\beta }^{\text{ initial }}])$ and $DDP_C([\varvec{\beta }_{\alpha }],[\varvec{{\hat{\beta }}}]) = \alpha DDP_C([\varvec{\beta }^{\text{ initial }}], [\varvec{{\hat{\beta }}}])$. In case of **LPCEO**, we can write $DEO_M([\varvec{\beta }_{\alpha }]) = \alpha DEO_M([\varvec{\beta }^{\text{ initial }}]) + (1-\alpha )\cdot \hat{\beta }\cdot \Delta$ where $\Delta$ depends on $[\varvec{{\hat{\beta }}}]$.

##### Configurations for baselines

 Similarly, we observe that all the baseline algorithms have some tunable parameters changing which we can generate different configurations with varying *DDP*s. These parameters are: *multiplicative covariance factor* $f \in [0,1]$ (Zafar et al.), *difference-bound* $\in [0,1]$ (Agarwal et al.), Lagrangian multiplier $\lambda \in [0,B_1]$ (Padala et al.) and primal-dual parameter $\delta \in [0,B_2]$ (Yang et al.). For each of these parameters, we divide its range into three equal parts and take the end points to generate four different configurations. For example, we take $f = 0,0.33,0.66,1$ to generate configurations of Zafar et al. The least $DDP_M$ configuration is attained at one of the end-points of the range of these parameters. For instance, it is attained at $f = 1$ for Zafar et al., *difference-bound* = 0 for Agarwal et al. etc.. However, we must reiterate that we cannot input any arbitrary configuration in these cases.

We also generate configurations from the equalized-odds implementation of the various baselines by tuning parameters namely : *DCCP parameters* ($\tau , \mu > 0$) (Zafar et al.), *difference-bound* (Agarwal et al.), Lagrangian multiplier (Padala et al.), varying random seeds (Cho et al., Romano et al., Hardt et al.), varying epochs (Mary et al.). We compare configuration-wise $DEO_M$ and accuracy of **LPCEO** with these baselines discussed in next section.

### Experimental evaluation

To demonstrate the effectiveness of our proposed approach, we deploy it across different datasets and compare it with the existing baseline algorithms.

#### Configuration based demographic parity

We first investigate if **LPC** is able to achieve a provided configuration. To this aim, given a dataset, we consider three different configurations. However, for a given configuration $[\beta ] = c$, the acceptance rates of all the subpopulations are chosen to be same (i.e., $\forall \beta _i \in [\beta ], \beta _i = c$). In Table 1, we report $\varepsilon$ ($DDP_C$), the minimum error (i.e., deviation from the given configuration $[\beta ]$) as achieved by **LPC** for various datasets and multiple sensitive attributes for both real-world and synthetic datasets. **LPC** achieves low $\varepsilon$ values for all the real-world datasets. For synthetic data, we observe an increase in $\varepsilon$ as the number of sensitive attributes increases for different values of $\beta$. The feasibility of the LP for larger number of constraints requires a larger value of $\varepsilon$, however, still **LPC** achieves low $\varepsilon$ even with ten sensitive attributes.

**Table 1:** $\varepsilon$ ($DDP_C$) that leads to feasibility of **LPC** for various datasets and acceptance rates ($\beta$).

Datasets	$\beta = 0.1$	$\beta = 0.25$	$\beta = 0.4$	Synthetic	$\beta = 0.1$	$\beta = 0.25$	$\beta = 0.35$
Adult	0.004	0.0025	0.0045	$k=2$	0.0005	0.0007	0.0004
COMPAS	0.002	0.002	0.0005	$k=6$	0.0006	0.0006	0.0007
Bank	0.0004	0.0005	0.0006	$k=10$	0.001	0.001	0.001
German	0.009	0.0025	0.008	$k=20$	0.1809	0.0236	0.0177

For Synthetic datasets results shown for different number of sensitive groups (*k*) and acceptance rates ($\beta$). In synthetic dataset each of the synthetic attributes are considered binary. So *k* = 20 implies that there are 20 sensitive attributes and in all 40 possible subpopulations.

#### Comparison with baselines ($DDP_M$)

We consider two setups for our experiments. In the first setup (**Minimum** $DDP_M$), given a dataset, we compare the minimum *DDP* and the corresponding accuracy achieved by the baseline algorithms and **LPCA**. For **LPCA**, we iterate over different configurations in the range [0, 1] (acceptance rates are same for all subpopulations) and consider the one for which the accuracy is maximized. In the second setup, we deploy **LPCA** across different configurations generated utilizing the method proposed in Sect. "Deriving a configuration". This allows us to demonstrate the stability of the results and makes for a fairer comparison.

##### Minimum $DDP_M$

We here present a comparative analysis of the lowest $DDP_M$ values and the corresponding accuracies achieved by the baselines and **LPCA** in Table 2. The lowest value is achieved by tuning the individual parameters as mentioned in the Sect. "Deriving a configuration". We find that **LPCA** outperforms almost all the algorithms (performing on multiple overlapping subpopulations) by one or two order of magnitude in terms of attaining minimum $DDP_M$. Among the baselines, Yang performs the best and produces the best result in the Bank Dataset. Since DELTR and Greedy-fair are implemented on single binary sensitive attribute, their least DDP values are better than that of **LPCA** in Table 2. Also for the case of $DDP_M$, the framework of Mehrotra is same as that of **LPCA** except the choice of *w*(*a*), hence we observe results close to that of **LPCA** . The accuracies are comparable across baselines, with **LPCA** performing marginally better. For **LPCA** the least $DDP_M$ obtained corresponds to $\alpha = 0, \hat{\beta } = 0.2, 0.05, 0.45$ and 0.7 for Adult, Bank, COMPAS and German datasets respectively.

##### Different configurations

 For each baseline algorithm, we generated four configurations by regulating a tunable parameter (Sect. "Deriving a configuration"). Subsequently, taking those four configurations as input, we run **LPCA** and obtain the results. We resort to this as any arbitrary configuration cannot be generated by baselines, and hence for fair comparison, results need to be generated at only feasible (for baselines) configurations.

For this particular set of four configurations, we also compute the average of the difference between accuracy, weighted precision and weighted recall and show it in Table 3. We can see that for most of the cases, **LPCA** performs better than the baselines (positive numbers). Only in two cases **LPCA**’s recall is worse. In all the instances, **LPCA** always performs better than its competitor in at least two of the three metrics. However, there is clearly no second best, showing the maturity of the field where several algorithms achieve similar performance. Note that ‘NA’ in Table 3 indicates that for all the four configurations, the average acceptance rate is lower (higher) than the natural acceptance rate of the lowest (highest) class. Noticeably, most of the algorithms fail to minimize $DDP_M$ keeping the average acceptance rate constant; we would also like to point that to assess the performance of an algorithm, a clear reporting of both $DDP_M$ and $\beta$ is needed.

**Table 2:** The *minimum* $DDP_M$ achieved by various baselines along with the corresponding test accuracies for various datasets.

Baseline	Adult		Bank		COMPAS		German	
	$DDP_M$	Accuracy	$DDP_M$	Accuracy	$DDP_M$	Accuracy	$DDP_M$	Accuracy
Zafar	0.1074	0.8357	0.0656	0.9027	0.0668	0.6559	0.0841	0.74
Agarwal	0.0711	0.8035	0.0335	0.9049	0.0251	0.6344	0.0895	0.75
Yang	0.018	0.7812	0.0069	0.8935	0.0356	0.5580	0.0655	0.7333
Padala	0.0658	0.8031	0.02	0.8735	0.0154	0.6256	0.0396	0.69
Mehrotra	0.0049	0.8061	0.0198	0.9044	0.0028	0.63	0.0025	0.7066
Madras	0.0332	0.8358	0.0403	0.9075	0.0403	0.6616	0.0534	0.7200
DELTR	0.0034	0.6529	**0.0048**	0.8291	0.0049	0.4981	0.0312	0.6066
Greedy-Fair	0.0004	0.56	0.0688	0.68	0.0014	0.5033	**0.0073**	0.4733
**LPCA**	**0.0049**	**0.8444**	0.008	**0.908**	**0.0009**	**0.6723**	0.0075	**0.7533**

Significant values are in bold.

In almost all the datasets, the least $DDP_M$ and the highest accuracy in that configuration is achieved by **LPCA**. The results of DELTR (ranking) and Greedy-fair (subset-selection) are obtained for single (binary) sensitive attribute their values and hence are not compared with other algorithms performing on multi-attribute case.

**Table 3:** Difference in average weighted precision (of higher acceptance class), weighted recall (of lower acceptance class) and overall accuracy over the 4 configurations, with **LPCA** for different datasets.

	Baseline	$\varvec{D_{Acc}}$	$\varvec{D_{Prec}}$	$\varvec{D_{Recall}}$	Baseline	$\varvec{D_{Acc}}$	$\varvec{D_{Prec}}$	$\varvec{D_{Recall}}$
Adult	Agarwal	0.0346	0.0306	-0.025	Madras	0.0002	0.0288	0.1416
	Zafar	0.0013	0.0677	0.0497	Yang	0.0347	0.221	NA
	Padala	0.0173	0.0669	NA	Mehrotra	0.0196	0.0349	0.1214
	DELTR	0.1849	0.5333	0.6093	Greedy-Fair	0.1716	0.3582	0.5372
Bank	Agarwal	0.0042	0.060	NA	Madras	0.0019	− 0.028	0.2695
	Zafar	0.0042	0.0664	NA	Yang	0.0043	0.0733	NA
	Padala	0.0119	0.0193	NA	Mehrotra	0.0024	0.0095	0.1222
	DELTR	0.083	0.5724	NA	Greedy-Fair	0.0904	0.578	0.4461
COMPAS	Agarwal	0.0039	NA	0.0102	Madras	0.0015	0.0078	0.0575
	Zafar	0.0013	0.0046	-0.008	Yang	0.0697	0.1103	0.1060
	Padala	0.0028	NA	0.0018	Mehrotra	0.0101	0.0168	-0.0035
	DELTR	0.1717	0.2804	0.1806	Greedy-Fair	0.14	0.0714	0.144
German	Agarwal	0.0232	0.0098	0.0377	Madras	0.0015	0.0078	0.0575
	Zafar	0.0199	0.0162	0.0022	Yang	0.0244	0.0158	0.0173
	Padala	0.055	0.0378	0.0417	Mehrotra	0.0300	0.0278	-0.0026
	DELTR	0.1434	NA	0.1044	Greedy-Fair	0.3278	0.0522	NA

Positive values mean **LPCA** is better. DELTR, Greedy-Fair and Madras perform on single binary sensitive attributes.

#### Comparison with baselines ($DEO_M$)

We perform configuration based comparison of $DEO_M$ with various baselines. For each of the baselines that can cater to equalized odds criteria of fairness, we find the configurations $[\varvec{\beta }]$ corresponding to their final output and use that $[\varvec{\beta }]$ in **LPCEO** to return output configurations $[\varvec{\beta '}]$ and compare $DEO_M$ of the baseline and that of **LPCEO**. Note that **LPCEO** ensures that $DDP_C([\varvec{\beta }], [\varvec{\beta '}]) \le \varepsilon$ for all the cases. We consider four different configurations for each baseline and observe that none of the baselines except Agarwal et al. and Yang et al. can cater to multiple overlapping sensitive attributes. Among the chosen baselines, the algorithms of Zafar et al., Romano et al. and Padala et al. can only deal with single binary sensitive attribute while the rest can cater to single sensitive attribute with multiple subpopulations. Thus, in our comparisons, we use binary and non-binary sensitive attributes according to the ability of the baselines. In Table 4, we show the average difference (over 4 configurations) of $DEO_M$ and accuracy between **LPCEO** and other baselines.

In the multiple overlapping subpopulations case, we observe that Agarwal et al. can achieve marginally better $DEO_M$ (at cost of accuracy) in the datasets except Adult whereas **LPCEO** performs better w.r.t $DEO_M$ when compared with Yang et al. in datasets other than Adult. In case of single sensitive attribute with multiple subpopulations, Mary et al. performs better on Adult whereas **LPCEO** performs better than Cho et al. and Hardt et al. in terms of $DEO_M$. In the case of single binary sensitive attribute, **LPCEO** performs better than Zafar et al, Padala et. al. and Romano et al. w.r.t. $DEO_M$. In almost all cases, **LPCEO** performs better in terms of test accuracy, although the improvement is modest.

#### Performance wrt $DDP_M$, $DEO_M$ and $\hat{\beta }$

##### Demographic parity

**Figure 3:**
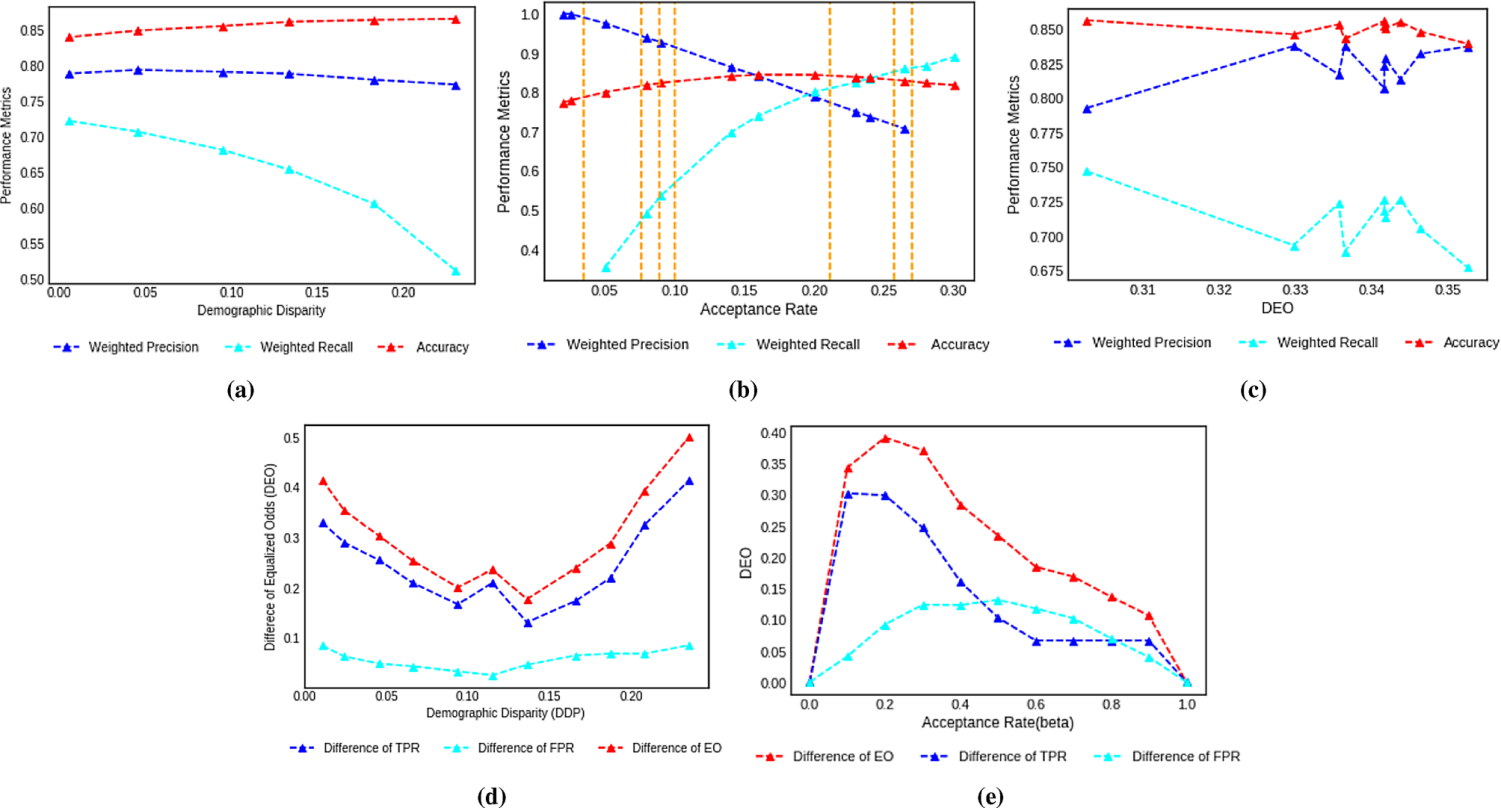
The variation of different performance metrics on configs generated by **LPCA** and **LPCEO** on Adult dataset with (**a**) increasing $DDP_M$ and fixed $\hat{\beta }=0.2$, (**b**) increasing $DEO_M$, (**c**) increasing $\hat{\beta }$. The seven vertical lines correspond to the initial configuration of acceptance rates of each of the seven subpopulations and fixed $\hat{\beta }=0.2$. The variation of $DEO_M$ on configurations generated by **LPCEO** with (**d**) increasing $DDP_M$ by varying $\alpha$ and $\hat{\beta =0.2}$, (**e**) increasing $\hat{\beta }$ and $\alpha = 0$.

 We perform experiments to show the way precision, recall, accuracy changes as we change the user tunable parameter $\alpha$ defined in Sect. "Deriving a configuration" and the desired acceptance rate (of all the subpopulations) $\hat{\beta } > 0$ to provide an idea about the relationship among these metrics and parameters. In Fig. 3a we observe the behavior of weighted precision, weighted recall and accuracy with varying $DDP_M$ on the configs generated by **LPCA** (as we vary $\alpha$ for Adult dataset). The acceptance rates of various classes output by the RF ([$\beta ^{\text{ RF }}$]) classifier is considered as $[{\beta }^{\text{ initial }}]$. In the plot we observe an increase in accuracy and decrease in precision and recall as the $DDP_M$ value increases. This happens because as $DDP_M$ increases, the system moves more towards $[\beta ^{\text{ initial }}]$, hence increasing the accuracy. Since higher classes begin to get a higher fraction of allocation, less confident points get a higher chance to be chosen, hence the dip in precision; the fall in recall can be explained similarly. In Fig. 3b we run **LPCA** on increasing values of $\hat{\beta }$ and observe that (a) the accuracy reaches a maxima at around $\hat{\beta } = 0.20$ that is due to the symmetry of this metric w.r.t. ‘natural’ average acceptance rate of the system, (b) as the value of $\hat{\beta }$ changes the precision/ recall curves go through seven event points corresponding to $\beta ^{\text{ initial }}_{j}$ of each class wherein as $\hat{\beta }$ increases, at each event point a particular class flips its participation from precision to recall calculation. Thus a temporary reverse trend was observed at those points.

##### Equalized odds

 Similar to the case of $DDP_M$, for a fixed $\hat{\beta }=0.2$ we vary $\alpha$ to generate configs with various $DEO_M$ in **LPCEO** and plot the corresponding weighted precision, recall and accuracy in Fig. 3c by taking the same $[{\beta }^{\text{ initial }}]$. We also plot in Fig. 3d the $DEO_M$ and the difference of *TPR* and *FPR* with varying $\alpha$, keeping $\hat{\beta } = 0.2$ and varying $\hat{\beta }$ keeping $\alpha = 0$. We observe that for a fixed $\hat{\beta } = 0.2$ as we vary $\alpha$, both $DEO_M$ and $DTPR_M$ are in general *non-monotonic* w.r.t. $DDP_M$ and reach a minimum at a particular $\alpha = \alpha _0 \in (0,1)$. Because of this non-monotonic relation the accuracy, weighted precision and recall also show an oscillating behavior when plotted against $DEO_M$, although the overall trends of the curves are similar to that of $DDP_M$ plot. In Fig. 3e, the value of $DEO_M$ attains a maximum at $\hat{\beta } = 0.2$, that is due to the fact that this value of $\hat{\beta }$ is the point of maximum accuracy and essentially corresponds to selecting a lot of people in a big population class (like ’male’); over less populated groups hence leading to larger *DTPR*.

**Table 4:** Average Difference (over 4 configurations) of $DEO_M$ and Accuracy between various baselines and **LPCEO**.

	Adult		Bank		COMPAS		German	
Baseline	$D_{DEO_M}$	$\varvec{D_{Acc}}$	$D_{DEO_M}$	$\varvec{D_{Acc}}$	$D_{DEO_M}$	$\varvec{D_{Acc}}$	$D_{DEO_M}$	$\varvec{D_{Acc}}$
Zafar	0.1239	0.0354	0.3199	0.2866	0.083	0.0197	0.0695	− 0.0022
Agarwal	0.0678	0.0332	− 0.0992	0.0068	− 0.0284	− 0.0181	0.0360	0.0183
Padala	0.06281	0.0282	NA	NA	0.0089	0.0085	0.0165	0.0078
Yang	− 0.081	0.0448	0.2817	0.0031	0.0893	− 0.0032	0.0649	0.0167
Romano	0.1175	0.0360	0.1430	0.015	− 0.0109	0.0052	0.0726	0.0092
Mary	0.0387	− 0.0048	0.0602	0.0652	0.0224	0.0227	0.041	0.0025
Cho	0.0883	0.005	NA	NA	0.0311	0.0167	NA	NA
Hardt	0.0389	0.0071	NA	NA	− 0.04	0.014	0.0003	0.0092

Positive values imply that **LPCEO** is performing better. Zafar, Padala and Romano can handle only single binary sensitive attributes. NA entries refer to a scenario in which the baseline is giving trivial classification as output (all 1’s or all 0’s) that results in $DEO_M = 0$ and hence **LPCEO** also attains the same accuracy and $DEO_M$ at that configuration.

#### Robustness

We further investigate whether the results obtained by the proposed algorithms are sensitive to train/test data splits. More specifically, in Table 5 we perform 10 random 70/30 splits of the data in two types of experiments and report the average $DDP_M, DEO_M$ and accuracy. For the case of **LPCA** for each dataset we fix a $\hat{\beta }$ with $\alpha = 0$ and report the average $DDP_M$ and accuracy. In the case of **LPCEO** for each dataset we fix a particular configuration $[\beta ]$ with $\alpha = 1$ and report the average $DEO_M$ and accuracy over the 10 random splits. In general, we observe that the statistics only change in the third digit after the decimal, if we take the average over 10 random splits and hence we do not show the results for other performance metrics. These results indicate that the proposed algorithms are robust and not sensitive to data splits.

**Table 5:** For **LPCA** the average values of accuracy and $DDP_M$ obtained for 10 random 70/30 splits are reported for $\alpha = 0$ and $\hat{\beta }= 0.2$ (Adult), $\hat{\beta }= 0.17$ (Bank), $\hat{\beta }= 0.47$ (COMPAS) and $\hat{\beta }= 0.84$ (German).

Dataset	**LPCA**		**LPCEO**	
	Accuracy	$DDP_M$	Accuracy	$DEO_M$
Adult	0.8387 ± 0.0028	0.0054 ± 0.002	0.8523 ± 0.0012	0.256 ± 0.0235
Bank	0.8905 ± 0.0017	0.0294 ± 0.0139	0.9134 ± 0.0006	0.4396 ± 0.0566
COMPAS	0.6535 ± 0.008	0.0013 ± 0.0008	0.6681 ± 0.0033	0.0.4193 ± 0.0046
German	0.764 ± 0.0227	0.0046 ± 0.0025	0.7533 ± 0.0057	0.1802 ± 0.0133

For **LPCEO** we fix $\alpha =1$ and a particular configuration [$\beta$] for every dataset and report the average $DEO_M$ and accuracy over 10 splits. The configurations used for the datasets for **LPCEO** are [$\beta$] = [0.2542, 0.1854, 0.2445, 0.1360, 0.2277, 0.1533, 0.1130] (Adult), [$\beta$] = [0.2468, 0.0592, 0.0611, 0.0887, 0.0477, 0.1851] (Bank), [$\beta$] = [0.3091, 0.5610, 0.4785, 0.3828] (COMPAS), [$\beta$] = [0.7330, 0.6170, 0.7137, 0.6153] (German).

#### Computational efficiency of methods

Next, we discuss the computational efficiency of different algorithms in terms of the average time taken (in seconds) for a fixed train-test split of the datasets, over 10 different values of the model parameter of the algorithms. Experiments are performed across *all the algorithms that can handle the multi-attribute case* for each dataset and the results are presented in Table 6. For Zafar, we use the parameter *multiplicative covariance factor*, for Agarwal *difference bound* and for Yang the parameters for the PLUGIN approach. The ranges of these values have been mentioned in Sect. "Deriving a configuration". Since these parameters regulate the output configuration of these algorithms, taking an average over these gives us an estimate of the effect of the strength of the fairness constraints on the running time of baselines. Similarly, for our methods **LPCA** and **LPCEO** we vary the target configuration $\hat{\beta } \in [0,1]$ with $\alpha = 0$ as the parameters in **LPCA** and **LPCEO** for all datasets. The time reported in this table (in seconds) is the addition of training and testing times. The experiments are performed on Intel Xeon CPU (2.2 GHz) with 13GB of RAM. Overall, **LPCA** and **LPCEO** achieve comparable performance in terms of computational costs with respect to the baseline methods.

**Table 6:** The average running time (in seconds) of baselines that can cater to the multiple sensitive attributes for the 4 datasets.

	Adult	Bank	COMPAS	German
Zafar	31.52	4.12	0.1	0.14
Agarwal	154.6	77.53	15.03	7.48
Yang	15.21	12.69	6.06	4.12
**LPCA**	40.9	49.05	4.8	0.59

	Adult	Bank	COMPAS	German
Agarwal	137.34	88.23	14.58	8.99
Yang	20.51	15.34	3.21	1.38
**LPCEO**	40.6	63.28	17.89	2.74

For the case of demographic parity Zafar, Agarwal and Yang can handle the multiple sensitive attributes whereas for Equalized Odds only Agarwal and Yang can cater to the multi-attribute case. Overall, **LPCA** and **LPCEO** achieve comparable performance in terms of computational costs.

#### Fair gerrymandering

The problem of fair gerrymandering proposed by Kearns et al.^54^ demonstrates that there could be large hidden subgroups in a particular data for which fairness may not naturally flow even if the overall system is fair. This problem, although mentioned by Zafar et al.^6^, was further explored in detail by Yang et al.^14^ who construct these gerrymandering subgroups and realize low $DDP_M$. For the case of two sensitive attributes (which is the case with most of our datasets) $S_1$ and $S_2$ containing $k_1$ and $k_2$ subpopulations respectively, the number of *gerrymandering* groups according to the definition of Yang is $k_1 \cdot k_2 + k_1 + k_2$. Thus, in all there are 17, 14, 8 and 8 gerrymandering groups for Adult, Bank, COMPAS and German datasets respectively of which only 13 and 10 have been considered for the Adult and Bank datasets respectively due to the small size of other groups. By regulating the tunable parameters of the algorithm, we are able to generate several configurations with varying $DDP_M$. These configurations are considered as input and **LPCEO** deployed to them. **LPCEO** is able to realize all the configurations, and we compare the performance of **LPCEO** in terms of accuracy and $DEO_M$ with Yang et al. and present the result in Table 7. We find that for the same $DDP_M$, **LPCEO** (**LPCA**) has a much better accuracy. The $DEO_M$ results are evenly distributed with **LPCEO** performing particularly well for COMPAS.

**Table 7:** Comparison of test accuracies, and $DEO_M$ between Yang et al.^14^ and **LPCEO** averaged over four configurations generated by Yang on various datasets.

Dataset	$DDP_M$	Yang		**LPCEO**	
		Accuracy	$DEO_M$	Accuracy	$DEO_M$
Adult	0.0683	0.7899	0.2716	0.828	0.3261
Bank	0.0632	0.9016	0.1323	0.9073	0.1272
COMPAS	0.1135	0.6564	0.113	0.6581	0.1366
German	0.1343	0.7498	0.2071	0.7683	0.2207

The number of gerrymandering groups for Adult, Bank, COMPAS and German datasets are 13, 10, 8 and 8 respectively.

#### Dependence on training distribution

As batch-wise post-processing frameworks, **LPCA** and **LPCEO** have a additional advantage of being invariant to training distribution of the sensitive attributes. This also allows for dealing with situations where the sensitive attributes are noisy which is often the case in real-world scenarios like online social media where the sensitive attributes are provided by users voluntarily. To illustrate, we consider an the Adult dataset and modify the ‘sex’ and ‘race’ attributes in the training data by randomly assigning their values to every element in the training data while keeping other dimensions/attributes unchanged. We also ensure that the accuracy of the base classifier (say RandomForest) largely remains unchanged. Noticeably, while all the algorithms mostly achieve similar levels of accuracy on the test dataset, none of them can maintain the same $DDP_M$ and $DEO_M$ after the training set is modified. This is because, unlike **LPCA** and **LPCEO**, all these algorithms assume that the distribution of sensitive attributes in the training and test data are the same and hence sensitive attribute information in the training set has a significant impact on the classification result of the test data. The detailed results are reported in Table 8. The DELTR algorithm, in which prediction is done based on a ranking function learnt on training set, is also affected by this modification, albeit to a less extent because it can only cater to single binary sensitive attribute. In the case of *DEO* some algorithms like Agarwal et al. and Hardt et al. are more affected by this experiment because they post-process and handle multiple subpopulations whereas in-processing algorithms like Yang et al., Padala et al., Zafar et al. etc. (some dealing with single binary sensitive case) are less affected.

**Table 8:** Test accuracy and $DDP_M$ and $DEO_M$ of various baselines on the Adult dataset with change in the ’sex’ and ’race’ attribute in the training data keeping the test data unchanged.

Baseline	Original train-test		Train modified	
	Accuracy	$DDP_M$	Accuracy	$DDP_M$
Zafar	0.831	0.21	0.8218	0.18
Padala	0.8031	0.0658	0.8346	0.1766
Agarwal	0.8035	0.0711	0.9056	0.052
Yang	0.7812	0.018	0.7985	0.1195
Madras	0.8323	0.0185	0.8505	0.1972
DELTR	0.6529	0.0034	0.6126	0.0044
**LPCA**	0.8335	0.019	0.8326	0.019

Baseline	Original train-test		Train modified	
	Accuracy	$DEO_M$	Accuracy	$DEO_M$
Zafar	0.811	0.23	0.805	0.29
Padala	0.8126	0.1633	0.8395	0.189
Agarwal	0.8543	0.1324	0.8617	0.3987
Yang	0.795	0.2564	0.795	0. 2581
Mary	0.8430	0.2787	0.8402	0.4333
Romano	0.8088	0.1612	0.8046	0.2239
Cho	0.8423	0.2176	0.8452	0.4073
Hardt	0.8396	0.2948	0.8645	0.4598
**LPCEO**	0.8637	0.1778	0.8637	0.1778

We have chosen our $DDP_M$ similar to Yang by tuning $\alpha , \hat{\beta }$ to show comparative results. The DELTR algorithm can only cater to single binary sensitive attribute.

## Conclusion

The primary contribution of this paper is in identifying the presence of a special but widespread batch-admission-like situation where batch classification is a natural operation. This decoding of an apparently obvious real-world setting helps us to design a simple LP-based algorithm that is being able to compete and perform better than sophisticated classification algorithms. We carefully generalize the definition of demographic parity and equalized odds for multiple sensitive attributes and analyze its theoretical computational complexity. These definitions help us to develop the LP framework which enables the generation of the desired configuration, be it expressed in terms of demographic disparity, average acceptance rate or a simple externally defined distribution of acceptance rates. Additional advantages of our configuration based LP framework includes - (i) ability to deal with multiple overlapping subpopulations, (ii) invariance to changes in sensitive attribute distribution in training data and (iii) applicability to related problems such as fair gerrymandering. In the future, it would be interesting to see how our framework can be applied to notions of fairness like counterfactual fairness, calibration, etc. which are quite different from independence and separation based notions of fairness (Supplementary Information).

### Supplementary Information

Supplementary Information.

## Data Availability

All the codes and datasets are available at https://github.com/alphaaccount/fair-batch-classification.
