# Supplementary material for: Ensuring generalized fairness in batch classification
Source: Sci Rep. 2023 Nov 2;13:18892. doi: 10.1038/s41598-023-45943-1 (PMC10622441; doi:10.1038/s41598-023-45943-1)
Supplement: Supplementary file 1 — Supplementary Information. [file 41598_2023_45943_MOESM1_ESM.pdf]

6 Supplementary Material

In this section we provide the details of the Tables 3, 4 and 7 present in the main paper. In addition, we provide a Table 20 that describes the applicability of baselines to various sensitive attributes.

6.1 Details of Table 3

In Table 3 we have presented the difference in accuracy, precision and recall. In the following Table 9 we present the details of the results of all the baselines on the chosen configurations on the three datasets. More specifically, we present the average weighted precision (of higher acceptance class), weighted recall (of lower acceptance class) and overall accuracy over the 4 configurations of all the baselines, for the different datasets. Thus, combining the results of Table 3 in the main paper, we can retrieve the values obtained by our algorithm **LPCA** on these configurations.

	**Baseline**	Accur.	Preci.	Recall	**Baseline**	Accur.	Preci.	Recall
Adult	Agarwal	0.8260	0.7551	0.5750	Madras	0.8456	0.7563	0.6526
	Zafar	0.8451	0.7617	0.6154	Yang	0.7937	0.6914	NA
	Padala	0.8209	0.7490	NA	Mehrotra	0.8174	0.7157	0.5099
	DELTR	0.8139	0.7761	0.6058	Greedy-Fair	0.8192	0.7647	0.6642
Bank	Agarwal	0.9047	0.6601	NA	Madras	0.9090	0.7929	0.4557
	Zafar	0.9047	0.6439	NA	Yang	0.9022	0.6579	NA
	Padala	0.8922	0.6476	NA	Mehrotra	0.9071	0.6507	0.4524
	DELTR	0.9071	0.6412	NA	Greedy-Fair	0.8917	0.661	0.4551
COMPAS	Agarwal	0.6443	NA	0.6445	Madras	0.6639	0.6862	0.5131
	Zafar	0.6597	0.6027	0.6460	Yang	0.5867	0.5860	0.3455
	Padala	0.6520	NA	0.6490	Mehrotra	0.6505	0.6489	0.5200
	DELTR	0.6613	0.6011	0.7999	Greedy-Fair	0.6131	0.6124	0.5285
German	Agarwal	0.7441	0.7985	0.8702	Madras	0.7233	0.8120	0.8928
	Zafar	0.7499	0.7932	0.8954	Yang	0.7505	NA	0.9101
	Padala	0.7166	0.7711	0.7999	Mehrotra	0.7199	0.7836	0.8785
	DELTR	0.7218	0.7846	NA	Greedy-Fair	0.7561	0.7915	NA

Table 9. Average weighted precision (of higher acceptance class), weighted recall (of lower acceptance class) and overall accuracy over the 4 configurations of all the baselines, for the different datasets.

	**Configuration**	Accuracy	Precision	Recall
Zafar	Config 1	0.8541	0.7672	0.6281
	Config 2	0.8303	0.7718	0.5913
	Config 3	0.8543	0.7454	0.6278
	Config 4	0.8415	0.7622	0.6142
Agarwal	Config 1	0.8421	0.728	0.5877
	Config 2	0.8131	0.7801	0.5801
	Config 3	0.8396	0.767	0.5711
	Config 4	0.8090	0.7452	0.561

	**Configuration**	Accuracy	Precision	Recall
Zafar	Config 1	0.9202	0.6344	NA
	Config 2	0.8953	0.6686	NA
	Config 3	0.9181	0.6299	NA
	Config 4	0.8849	0.6425	NA
Agarwal	Config 1	0.9184	0.6406	NA
	Config 2	0.9197	0.6752	NA
	Config 3	0.8842	0.6503	NA
	Config 4	0.8963	0.6741	NA

Table 10. The accuracy, precision and recall of individual configuration whose average has been presented in Table 9 for the algorithms of Zafar and Agarwal for Adult (first table) and Bank (second table) datasets.

Next, we present the configurations of different baselines that have been used to get the results of Table 3 in the main paper. We only mention the configs of the baselines of Zafar et al. and Agarwal et al. for the three datasets and avoid the details of the other algorithms for a concise and tidy presentation. These configs are described in Tables 12, 13, 14 and 15. The accuracy, precision and recall of these configurations have been depicted in Table 10 and Table 11 for the four datasets.

6.2 Details of Table 4

Here we first present the average DEO_M and accuracy of various baselines over the chosen configurations as used in Table 4. Then analogous to Table 10 and 11, we present the values of DEO_M and accuracy for the four configurations of Zafar and Agarwal in Table 16 and also the DEO_M and accuracy for each configuration in Tables 17 and 18 for four datasets.

	Configuration	Accuracy	Precision	Recall
Zafar	Config 1	0.7682	0.8064	0.8715
	Config 2	0.725	0.7972	0.9006
	Config 3	0.7483	0.766	0.8933
	Config 4	0.7579	0.8029	0.916
Agarwal	Config 1	0.7367	0.7953	0.8953
	Config 2	0.7653	0.8255	0.8677
	Config 3	0.7517	0.7865	0.8668
	Config 4	0.7226	0.7865	0.8508

	Configuration	Accuracy	Precision	Recall
Zafar	Config 1	0.657	0.5914	0.6485
	Config 2	0.6479	0.5955	6707
	Config 3	0.6488	0.5934	0.6281
	Config 4	0.6849	0.6303	0.6365
Agarwal	Config 1	0.6594	NA	0.6549
	Config 2	0.6518	NA	0.6191
	Config 3	0.6174	NA	0.6609
	Config 4	0.6484	NA	0.6429

Table 11. The accuracy, precision and recall of individual configuration whose average has been presented in Table 9 for the algorithms of Zafar and Agarwal for German (first table) and COMPAS (second table) datasets.

	Gender		Race				
	Configurations of **Zafar et al.**[6]						
	Male	Female	White	Black	Asian	Am-Ind	others
Adult	0.2056	0.1196	0.1852	0.0991	0.2698	0.1	0.0608
	0.1791	0.1386	0.1716	0.1040	0.2400	0.1066	0.0782
	0.1407	0.1402	0.1415	0.1131	0.2178	0.14	0.0608
	0.1098	0.1567	0.1245	0.1106	0.1856	0.1466	0.0782
	Configurations of **Agarwal et al.**[5]						
	Male	Female	White	Black	Asian	Am-Ind	others
	0.2591	0.0730	0.2101	0.0893	0.3069	0.0933	0.0347
	0.2556	0.0766	0.2080	0.1016	0.2871	0.0933	0.0434
	0.1961	0.1267	0.1796	0.1401	0.1534	0.1333	0.0695
	0.1754	0.1421	0.1660	0.1647	0.1534	0.1400	0.1043

Table 12. The configurations for Adult dataset generated from Zafar et al. and Agarwal et al. for comparison with **LPCA**. The DDP_M of the configs is in descending order from top to bottom for each algorithm.

6.3 Details of Table 7

In Table 19, we present the DDP_M, DEO_M and accuracy of individual configuration whose average has been presented in Table 7 for the algorithms of Yang and **LPCEO** for Adult (first table) and Bank (second table) datasets. Since the same configuration is used to compute the DEP_M and accuracy of both algorithms, the DDP_M values of are the same for both for the corresponding configuration.

	Age		Marital Status		
	Configurations of **Zafar et al.**[6]				
	age<25 or age>60	25≤age≤60	Married	Single	Divorced
Bank	0.2158<br>0.1278<br>0.0967<br>0.1070	0.0556<br>0.0562<br>0.0560<br>0.0578	0.0599<br>0.0571<br>0.0553<br>0.0571	0.0782<br>0.0720<br>0.0682<br>0.0743	0.0443<br>0.0429<br>0.0472<br>0.0414
	Configurations of **Agarwal et al.**[5]				
	age<25 or age>60	25≤age≤60	Married	Single	Divorced
	0.0777<br>0.0794<br>0.0725<br>0.0742	0.0524<br>0.0517<br>0.0513<br>0.0513	0.0501<br>0.0493<br>0.0497<br>0.0497	0.0738<br>0.0729<br>0.0649<br>0.0629	0.0240<br>0.0254<br>0.0349<br>0.0407

Table 13. The configurations for Bank dataset generated from Zafar et al. and Agarwal et al. for comparison with **LPCA**. The DDP_M of the configs is in descending order from top to bottom for each algorithm.

	Race		Gender	
	Configurations of **Zafar et al.**[6]			
	White	Black	Male	Female
COMPAS	0.3605<br>0.3862<br>0.4102<br>0.4134	0.5093<br>0.4854<br>0.4802<br>0.4802	0.4738<br>0.4547<br>0.4619<br>0.4603	0.3611<br>0.4135<br>0.4166<br>0.4290
	Configurations of **Agarwal et al.**[5]			
	White	Black	Male	Female
	0.4070<br>0.4278<br>0.4615<br>0.4967	0.5208<br>0.5020<br>0.5156<br>0.5218	0.4841<br>0.4825<br>0.5000<br>0.5095	0.4444<br>0.4351<br>0.4722<br>0.5216

Table 14. The configurations for COMPAS dataset generated from Zafar et al. and Agarwal et al. for comparison with **LPCA**. The DDP_M of the configs is in descending order from top to bottom for each algorithm.

	Race		Gender	
	Configurations of **Zafar et al.**[6]			
	Age>25	Age≤ 25	Male	Female
	0.8324	0.8429	0.8376	0.8272
	0.688	0.7339	0.7339	0.7431
German	0.8204	0.7918	0.8163	0.7959
	0.6	0.8545	0.7272	0.8
	Configurations of **Agarwal et al.**[5]			
	Age>25	Age$\leq$25	Male	Female
	0.8219	0.7958	0.8167	0.8167
	0.7155	0.7614	0.7798	0.7522
	0.8081	0.8040	0.8204	0.8081
	0.6727	0.6909	0.7272	0.7272

Table 15. The configurations for German dataset generated from Zafar et al. and Agarwal et al. for comparison with **LPCA**. The DDP_M of the configs is in descending order from top to bottom for each algorithm.

	Adult		Bank		COMPAS		German	
**Baseline**	DEO_M	Accuracy	DEO_M	Accuracy	DEO_M	Accuracy	DEO_M	Accuracy
Zafar	0.235	0.8122	0.42	0.5896	0.21	0.645	0.22	0.7722
Agarwal	0.2523	0.7528	0.1879	0.9083	0.1012	0.6666	0.1955	0.7324
Padala	0.1633	0.8126	NA	NA	0.1355	0.6275	0.2238	0.7333
Yang	0.2564	0.795	0.2493	0.9094	0.4087	0.6862	0.2701	0.7333
Romano	0.1544	0.8015	0.3358	0.8987	0.3004	0.6454	0.169	0.7466
Mary	0.2787	0.8430	0.1467	0.8944	0.2571	0.6454	0.1977	0.7583
Cho	0.2176	0.8423	NA	NA	0.3974	0.6742	NA	NA
Hardt	0.2301	0.8012	NA	NA	0.0452	0.6426	0.1599	0.73

Table 16. Average DEO_M and Accuracy of various baselines over the chosen configs. NA entries refer to a scenario in which the baseline is giving trivial classification as output (all 1's or all 0's) that results in $DEO_M = 0$. To be read together with Table 4.

	**Configuration**	DEO_M	Accuracy
Zafar	Config 1	0.249	0.7927
	Config 2	0.2524	0.8004
	Config 3	0.2153	0.831
	Config 4	0.2232	0.8245
Agarwal	Config 1	0.2777	0.778
	Config 2	0.2345	0.7483
	Config 3	0.2445	0.7385
	Config 4	0.2523	0.7462

	**Configuration**	DEO_M	Accuracy
Zafar	Config 1	0.4036	0.9067
	Config 2	0.4143	0.6724
	Config 3	0.4443	0.6771
	Config 4	0.4176	0.7021
Agarwal	Config 1	0.1786	0.888
	Config 2	0.1873	0.9181
	Config 3	0.2123	0.9265
	Config 4	0.1731	0.9005

Table 17. The accuracy and DEO_M of individual configuration whose average has been presented in Table 16 for the algorithms of Zafar and Agarwal for Adult (first table) and Bank (second table) datasets.

	Configuration	DEO_M	Accuracy
Zafar	Config 1	0.2106	0.7666
	Config 2	0.2144	0.7938
	Config 3	0.2457	0.7524
	Config 4	0.2091	0.7757
Agarwal	Config 1	0.1707	0.7199
	Config 2	0.2003	0.7606
	Config 3	0.1997	0.7377
	Config 4	0.2111	0.7304

	Configuration	DEO_M	Accuracy
Zafar	Config 1	0.2136	0.6517
	Config 2	0.2316	0.6672
	Config 3	0.2079	0.6361
	Config 4	0.1867	0.6248
Agarwal	Config 1	0.1156	0.6522
	Config 2	0.1182	0.6933
	Config 3	0.0893	0.6569
	Config 4	0.0815	0.6569

Table 18. The accuracy and DEO_M of individual configuration whose average has been presented in Table 16 for the algorithms of Zafar and Agarwal for German (first table) and COMPAS (second table) datasets.

	Configuration	DDP_M	Accuracy	DEO_M
Yang	Config 1	0.0468	0.7675	0.2775
	Config 2	0.0612	0.8119	0.2753
	Config 3	0.0898	0.7943	0.2451
	Config 4	0.0752	0.7857	0.2882
**LPCEO**	Config 1	0.0468	0.8071	0.3288
	Config 2	0.0612	0.8450	0.3164
	Config 3	0.0898	0.8419	0.3083
	Config 4	0.0752	0.8178	0.3507

	Configuration	DDP_M	Accuracy	DEO_M
Yang	Config 1	0.0645	0.9235	0.1536
	Config 2	0.0736	0.9041	0.1418
	Config 3	0.0367	0.8785	0.1164
	Config 4	0.0778	0.9	0.1173
**LPCEO**	Config 1	0.0645	0.6406	0.9128
	Config 2	0.0736	0.6752	0.8827
	Config 3	0.0367	0.6503	0.9232
	Config 4	0.0778	0.6741	0.9103

Table 19. The DDP_M, DEO_M and accuracy of individual configuration whose average has been presented in Table 7 for the algorithms of Yang and **LPCEO** for Adult (first table) and Bank (second table) datasets.

	Adult		Bank		COMPAS		German	
**Baseline**	Eq. Odds	Dm. Parity	Eq. Odds	Dm. Parity	Eq. Odds	Dm. Parity	Eq. Odds	Dm. Parity
Zafar	Gender	Gender, Race	Age	Age, MS	Race	Race, Gender	Age	Age, Gender
Agarwal	Gender, Race	Gender, Race	Age, MS	Age, MS	Race, Gender	Race, Gender	Age, Gender	Age, Gender
Padala	Gender	Gender, Race	Age	Age, MS	Race	Race, Gender	Age	Age, Gender
Yang	Gender, Race	Gender, Race	Age, MS	Age, MS	Race, Gender	Race, Gender	Age, Gender	Age, Gender
Romano	Gender	-	Age	-	Race	-	Age	-
Mary	Race	-	MS	-	Race	-	Age	-
Cho	Race	-	MS	-	Race	-	Age	-
Hardt	Race	-	MS	-	Race	-	Age	-
Mehrotra	-	Gender, Race	-	Age, MS	-	Race, Gender	-	Age, Gender
DELTR	-	Gender	-	Age	-	Race	-	Age
Greedy-fair	-	Gender	-	Age	-	Race	-	Age

Table 20. The list of sensitive attributes in various datasets which we have used for comparative analysis with different baselines. The list depends on the ability of the baseline to handle single (binary/ non-binary) and multiple overlapping subpopulations e.g. Zafar can handle single binary sensitive attribute in case of EO, hence 'Gender' is taken in case of Adult dataset.
